# Active Compound of *Pharbitis* Semen (*Pharbitis nil* Seeds) Suppressed KRAS-Driven Colorectal Cancer and Restored Muscle Cell Function during Cancer Progression

**DOI:** 10.3390/molecules25122864

**Published:** 2020-06-22

**Authors:** Jisu Song, Heejung Seo, Mi-Ryung Kim, Sang-Jae Lee, Sooncheol Ahn, Minjung Song

**Affiliations:** 1 Department of Food Biotechnology, School of Medical and Life Science, Silla University, Busan 46958, Korea; jisu632@naver.com (J.S.); ssk4623@naver.com (H.S.); haha7kmr@silla.ac.kr (M.-R.K.); sans76@silla.ac.kr (S.-J.L.); 2 Department of Medical Science, School of Medicine, Pusan National University, Yangsan 50612, Korea; 3 Department of Cogno-Mechatronics Engineering, College of Nanoscience & Nanotechnology, Pusan National University, Busan 46241, Korea; 4 WT-MRC Institute of Metabolic Science, University of Cambridge, Cambridge CB20QQ, UK

**Keywords:** *Pharbitis nil*, colorectal cancer, KRAS, muscle function

## Abstract

Kirsten rat sarcoma viral oncogene homolog (KRAS)-driven colorectal cancer (CRC) is notorious to target with drugs and has shown ineffective treatment response. The seeds of *Pharbitis nil,* also known as morning glory, have been used as traditional medicine in East Asia. We focused on whether *Pharbitis nil* seeds have a suppressive effect on mutated KRAS-driven CRC as well as reserving muscle cell functions during CRC progression. Seeds of *Pharbitis nil* (*Pharbitis* semen) were separated by chromatography and the active compound of *Pharbitis* semen (PN) was purified by HPLC. The compound PN efficiently suppressed the proliferation of mutated KRAS-driven CRC cells and their clonogenic potentials in a concentration-dependent manner. It also induced apoptosis of SW480 human colon cancer cells and cell cycle arrest at the G2/M phase. The CRC related pathways, including RAS/ERK and AKT/mTOR, were assessed and PN reduced the phosphorylation of AKT and mTOR. Furthermore, PN preserved muscle cell proliferation and myotube formation in cancer conditioned media. In summary, PN significantly suppressed mutated KRAS-driven cell growth and reserved muscle cell function. Based on the current study, PN could be considered as a promising starting point for the development of a nature-derived drug against KRAS-mutated CRC progression.

## 1. Introduction

Colorectal cancer (CRC) is the third most diagnosed cancer worldwide. Recent statistics showed CRC caused 832,000 deaths and nearly 1.6 million new cases were diagnosed [1]. Among the various CRCs, Kirsten rat sarcoma viral oncogene homolog (KRAS) genetic mutation driven-CRC are known to be difficult to target, and prone to drug-induced side effects [2,3]. Mutated KRAS proteins cause persistent activation of GTP, which endlessly send signals to the RAS/extracellular signal–regulated kinases (RAS/ERK) or protein kinase B/mammalian target of rapamycin (AKT/mTOR) cell regulation pathways [2]. ERK or mTOR proteins activate transcription factors involved in cell death, cell cycle progression, and transcription [2,4]. To inhibit RAS activation, antibody therapy was developed to target EGFR, such as Cetuximab, but various drug-resistance issues were documented [5,6,7]. Therefore, novel medicinal substances with effective anti-cancer properties are needed and plant-derived foods or medicinal plants can offer potential means. 

Several natural products have exhibited anti-CRC activity. For example, Danshen (*Salvia miltiorrhiza*) improved the survival rate of patients with colorectal cancer [8]. Triterpenoids from *Rhus chinensis* Mill [9], protopine from *Nandina domestica* [10], Paris saponin VII from *Trillium tschonoskii* [11] and protein hydrolysates from Fenugreek (*Trigonella foenum graecum)* [12] suppressed the proliferation and induced apoptosis of CRC cells. Additionally, rosemary (*Rosmarinus officinalis*) inhibited CRC growth by inducing necrosis [13]. However, limited studies have examined the effects of natural extracts targeting mutated KRAS-driven CRC and none of these studies tested the efficacy of these extracts on muscle function. In our previous study, *Cordyceps militaris* germinated soybeans suppressed mutated KRAS-driven CRC via the RAS/ERK pathway [14]. *Phellinus linteus* grown on germinated brown rice increased the sensitivity of Cetuximab to inhibit KRAS-CRC progression both in vitro and in vivo [15]. Although the two natural compounds have anti-CRC activities, more medicinal substances are needed to target the notorious and drug-resistant nature of mutated KRAS-driven CRC. 

Morning glory (*Pharbitis nil*) is a well-known ornamental plant, and its seeds have been used in traditional medicine to treat various cancers and inflammatory diseases in Korea, China, and Japan [16]. Recent studies have demonstrated its suppressive effects on breast cancer [17], lung cancer [18] and gastric cancer cells [19]. In addition, the seed of *Pharbitis nil* is the main constitute of the approved drug, DA-9701 (Motilitone) [20]. DA-9701 is a botanical drug for treating functional dyspepsia and composed of the seed of *Pharbitis nil* and *Corydalis* tuber. Therefore, we anticipated its safety on human and can be adapted for therapies in a relatively short timeframe if proven its efficiency. In view of its anticancer effects, we considered the active compound of *Pharbitis* semen (PN) may inhibit the proliferation and progression of mutated KRAS-driven CRC without inducing serious side effects.

In addition to the suppressive effect of PN on CRCs, we evaluated the effect of compound PN on muscle cell function. During the course of CRC, cachexia, a complex syndrome showing severe muscle weight loss, is often accompanied with CRC progression and worsened treatment therapies [21,22,23]. Therefore, treating cachexia becomes an important combinational therapy in the treatment of CRCs. We mimicked cancer-associated environment with conditioned media and evaluated the effect of PN on muscle cells. 

In the present study, the anticancer activity of PN was investigated by examining its anti-proliferation and clonogenic features in five different CRC cell models. Cell cycle and apoptotic analyses were performed, and the changes in RAS/ERK and AKT/mTOR pathways were investigated. Finally, we assessed the effect of the compound PN on muscle cell proliferation and function.

## 2. Results and Discussion

### 2.1. PN Suppressed Colorectal Cancer Cell Progress

**Figure 1:**
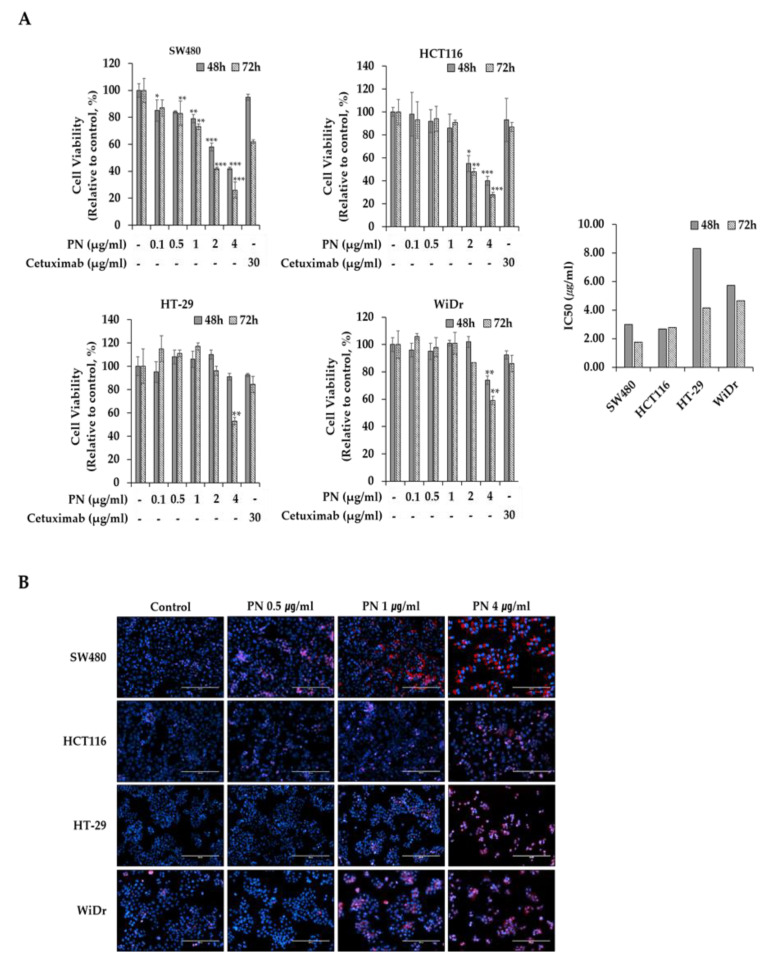
(**A**) PN suppressive effect on KRAS-mutated colorectal cancer cells of SW480 (KRAS^G12V^) and HCT116 (KRAS^G13D^), and KRAS-wild types CRCs of HCT116 and WiDr. Cells were treated for 48 and 72 h under PN treatment (0, 0.1, 0.5, 1, 2 and 4 µg/mL) and Cetuximab (30 µg/mL). IC_50_ values are calculated. Results are presented as means ± S.D. of three independent experiments. * *p* < 0.05, ** *p* < 0.01, *** *p* < 0.001. (**B**) Representative colorectal cancer cell images under PN treatment (0, 0.5, 1 and 4 µg/mL). Blue represents the DAPI-stained cell nuclei, and the propidium iodide-stained dead cells are red. Scale bar = 500 μm. DAPI, 4′,6-diamidino-2-phenylindole; PI, propidium iodide.

To investigate the anti-proliferative effects of PN on mutated KRAS-driven CRC, KRAS mutated SW480 and HCT116 cells as well as KRAS-wild type (WT) HT29 and WiDr were evaluated. Cells were treated with PN at 0, 0.1, 0.5, 1, 2, or 4 µg/mL and incubated for 48 or 72 h in triplicate. Cetuximab which has been used to treat mutated KRAS-driven CRCs was used as a control. Figure 1A summarized the proliferation profiles. PN inhibited cell growth in a concentration dependent manner in mutated KRAS-driven cell lines at 48 and 72 h. At 72 h, SW480 viability was decreased to 41.6 ± 1.0% when treated with PN at 2 µg/mL and to 26.0 ± 4.5% when treated with 4 µg/mL PN. When compared to control drug of Cetuximab, PN showed higher suppressive effect from 2 µg/mL concentration. Data also indicated that KRAS mutated cell lines responded more sensitively on PN treatment compared to KRAS wild type cells of HT-29 and WiDr. In KRAS wild type cells, PN treatment did not show statistical decrease on proliferation at 48 h. The IC_50_ values of KRAS mutated cells at 72 h are 1.74 and 2.78 µg/mL on SW480 and HCT116 cells, whereas they are 4.14 and 4.46 µg/mL on KRAS WT cells of HT29 and WiDr cells. IC_50_ values of KRAS mutated cells are relatively low compared to other natural extract such as pogostone (HCT116: 18.7 ± 1.93 µg/mL) [24].

Cells were stained with DAPI and PI for live and dead cell detection. Cell nuclei were stained with DAPI (blue) which represent all cells, and dead cells were stained with PI (red). Most cells in control group were stained with DAPI and little PI staining after 48 h of cultivation indicating most cells are alive (Figure 1B). In contrast, cells treated with PN began to appear dead PI stained cells from 0.5 µg/mL. 

**Figure 2:**
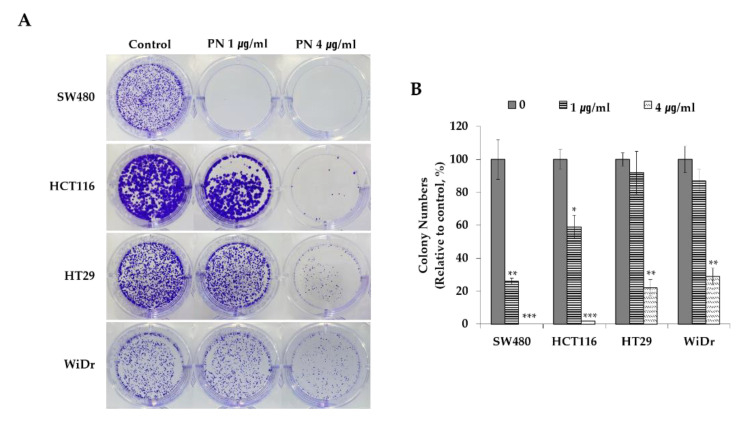
(**A**) Clonogenic potential after the PN treatment (0, 1 and 4 µg/mL) on KRAS-mutated colorectal cell lines of SW480 (KRAS^G12V^) and HCT116 (KRAS^G13D^) and KRAS-WT cells of HT29 and WiDr. (**B**) Relative colony numbers upon the PN treatment were calculated compared to the control. Results are presented as means ± S.D. of three independent experiments. * *p* < 0.05, ** *p* < 0.01, *** *p* < 0.001.

By assessing clonogenic potentials, we evaluated if PN inhibited tumorogeneity. As shown in Figure 2, PN dose-dependently suppressed colony formation by the KRAS mutated cell lines SW480, and HCT116. For example, PN treatment at 1 µg/mL suppressed SW480 cell colony formation to 26%. Surprisingly, almost no colony formation was observed by KRAS mutated CRCs treated with 4 µg/mL of PN. In contrast, HT29 and WiDr colonies are still visible even at the highest concentration. These findings show that PN sensitively suppress proliferation rate and tumor forming abilities in the long-term aspects of mutated KRAS-driven CRC cells. Further mechanistic and molecular studies were performed with SW480 cells at PN concentrations ranging from 0.1 to 4 µg/mL. 

Seeds of *Pharbitis nil* contains several bioactive compounds such as polysaccharides [16], pharbitin (resin glycosides) [25], anthocyanins, diterpenoids [26], triterpene saponins [27], pharbilignan C [17], neolignane, and monoterpene glycosides [28]. Pharbilignan C has been reported to induce human breast cancer cell apoptosis via the mitochondria-medicated intrinsic pathway [17], and lignans exhibited anti-inflammatory and anti-cancer activities [29]. Thus, the observed suppressive effects of PN on CRC cell-lines may have been due to the presence of these functional compounds. 

### 2.2. PN Induced Apoptosis and Cell Cycle Arrest in the G2/M Phase

**Figure 3:**
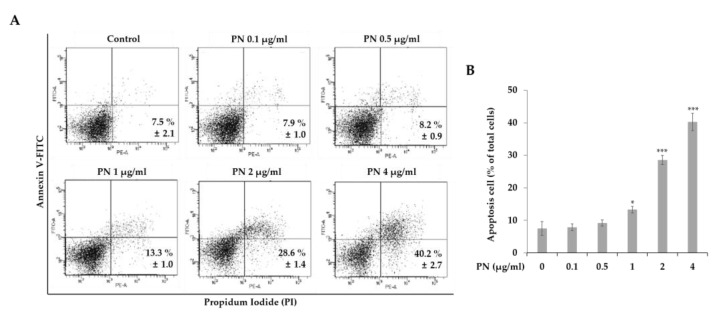
(**A**) Flow cytometric apoptosis images PN treatment using Annexin V-FITC/PI staining. SW480 cells were stained with Annexin V-FITC/PI upon PN treatment (0, 0.1, 0.5, 1, 2, and 4 µg/mL). (**B**) The percentage of apoptotic cells upon the PN treatment were compared. Asterisks (*) indicate statistical differences compared to untreated control (n = 3). * *p* < 0.05, ** *p* < 0.01, *** *p* < 0.001; Student’s *t* test. Statistical differences among the experimental groups were confirmed via one-way ANOVA test. FITC, fluorescein isothiocyanate; PI, propidium iodide.

To investigate the mechanism of cell death in mutated KRAS-driven human colorectal cell lines induced by PN, we analyzed apoptosis and cell cycles. For detecting apoptotic cells, Annexin V-FITC and PI staining was used. Annexin V binds specifically to a phosphatidylserine residue that is externalized by cells undergoing apoptosis. As seen in Figure 3, PN substantially increased the percentage of apoptotic cells in a concentration-dependent manner. Early (Annexin V FITC^+^/PI^−^) and late (Annexin V FITC^+^/PI^+^) apoptosis counts were increased up to 28.6 ± 1.4% and 40.2 ± 2.7% at 2 or 4 µg/mL PN treatment for 48 h, respectively. These observations suggest PN inhibits CRC proliferation by inducing apoptosis. 

**Figure 4:**
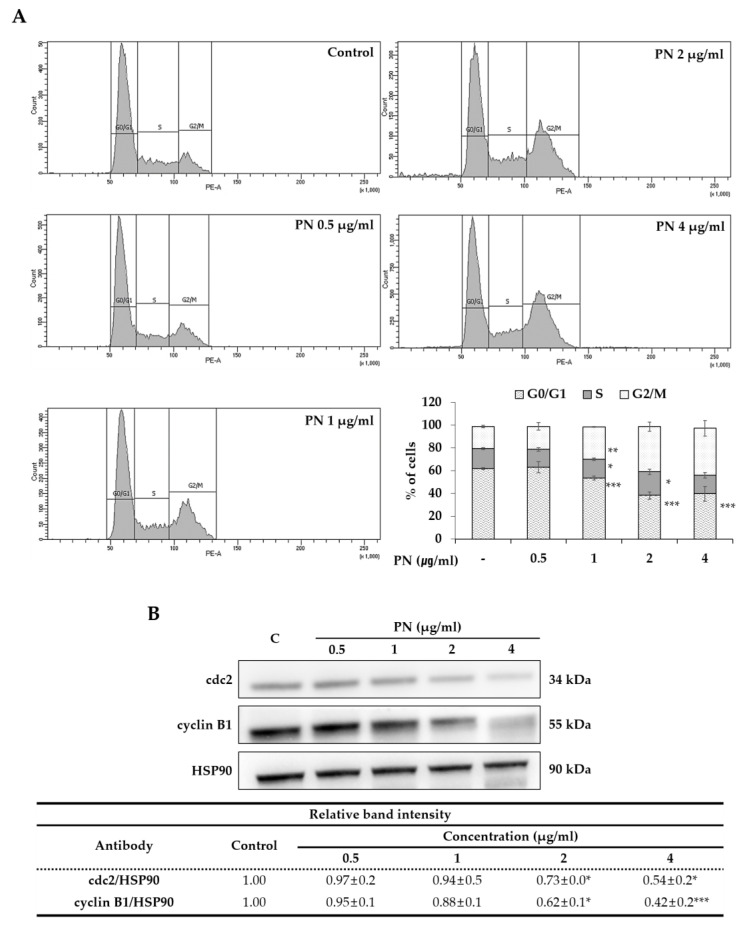
(**A**) Cell cycle analysis upon PN treatment (0, 0.5, 1, 2, and 4 µg/mL). Images represent the SW480 cell response under PN treatment. Percentages in each cell cycle phase (G0/G1, S, G2/M) are calculated and G2/M phase are increased as concentration dependent manner. Asterisks indicates statistical differences compared to untreated control through Student’s *t*-test (* *p* < 0.05, ** *p* < 0.01, *** *p* < 0.001). (**B**) Expression of G2/M phase related proteins of cdc2 and cyclin B1 upon PN treatment. HSP90 was used as a loading control and the protein intensity was analyzed in triplicate. The ratios of cdc2/HSP90 and cyclin B1/HSP90 were calculated and analyzed by Student *t*-test * *p* < 0.05, ** *p* < 0.01, *** *p* < 0.001.

Cell cycle is progressed into four phases: gap1 (G1), DNA synthesis (S), gap2 (G2) and mitosis (M). Arresting cancer cells at a certain stage are often viewed as therapeutic targets [30]. The cell cycle after exposing SW480 cells at concentrations of 0.5–4 µg/mL of PN was investigated. As seen in Figure 4A, cell cycle analysis showed PN concentration-dependently induced accumulation in the G2/M phase and a concomitant reduction in the G0/G1 phase. For example, the proportions of cells in the G2/M phase after treatment with 1 or 4 µg/mL of PN were 28.5 ± 0.3 % and 41.2 ± 6.7 %, respectively, which demonstrated PN inhibited cell growth by arresting cells in the G2/M phase. Then, we investigated expression levels of the cdc2 and cyclin B1, which are proteins for cell cycle progression from G2/M to G0/G1. Indeed, western blot images revealed that PN concentration-dependently reduced the levels of both proteins (Figure 4B).

PN was found to arrest cells in the G2/M phase to reduce the number of cells in the G0/G1 phase (Figure 4), therefore less cells enter into mitosis. This is interesting in that most of the cell cycle arrests caused by natural extracts reported to date have occurred in the G0/G1 or G2/M stages. [21,31,32]. For example, pogostone induced G0/G1 arrest in a KRAS mutated HCT116 cell-line [24]. 

### 2.3. Inhibition of the AKT/mTOR Pathway Enhanced PN-Induced Cell Death

**Figure 5:**
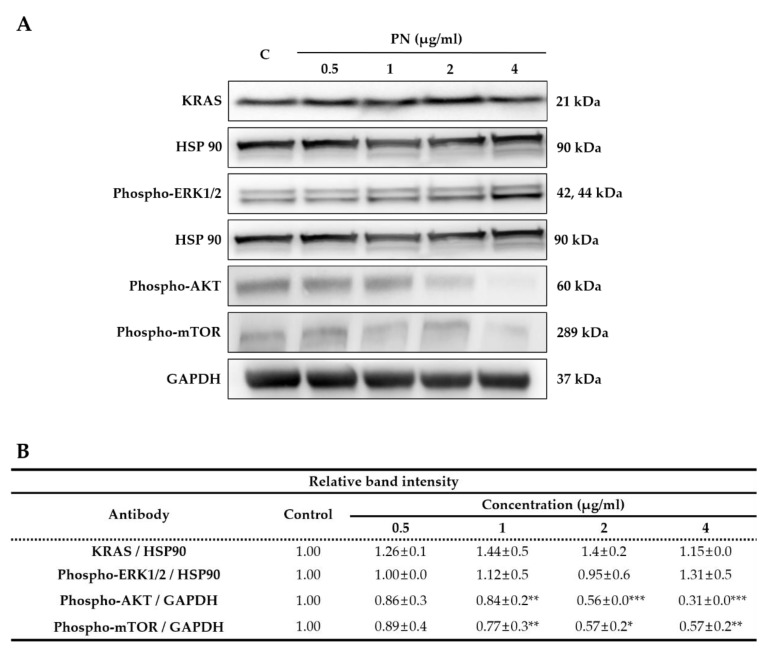
(**A**) Representative western blot images of KRAS, HSP90, phospho-p42/44 MAPK (phospho-ERK1/2), phospho-AKT, phospho-mTOR protein expression in SW480 cells treated with the compound PN (0, 0.5, 1, 2 and 4 µg/mL). (**B**) The ratios of KRAS/HSP90, phospho-ERK1/2/HSP90, and phospho-AKT/GAPDH, phospho-mTOR/GAPDH were calculated and compared to the control. (N= 3; mean SEM, * *p* < 0.05, ** *p* < 0.01, *** *p* < 0.001; Student’s *t* test).

Mutated KRAS-driven CRC signals are through the ERK/MEK or AKT/mTOR pathways [3,4]. During mutated KRAS-driven CRC progression, mutant RAS constitutively activates ERK/MEK or AKT/mTOR phosphorylation, and as a result promotes cancer cell proliferation. To determine whether SW480 growth inhibition by PN is regulated by RAS/ERK or AKT/mTOR pathways, cells are treated with PN for 48 h and protein levels of KRAS, p-ERK, p-AKT, and p-mTOR were assessed by western blot. As shown in Figure 5, PN significantly and concentration-dependently suppressed p-AKT and p-mTOR phosphorylation. For example, the expression of p-AKT was reduced to 0.31 in PN 4 µg/mL treated cells as compared with 1.0 in the non-treated control. The level of KRAS and ERK phosphorylation was similar in all groups as observed in Figure 5. These results demonstrated that PN suppressed CRC progression predominantly via the AKT/mTOR pathway. The AKT/mTOR and ERK/MEK pathways are known to be major regulatory signaling pathways that relate to CRC cell proliferation, metabolism, and survival [2,4,33]. Our observations suggest that the antitumor effect of PN is caused by reductions in the phosphorylations of AKT and mTOR.

### 2.4. PN Restores Muscle Cell Function during Cancer Progression

**Figure 6:**
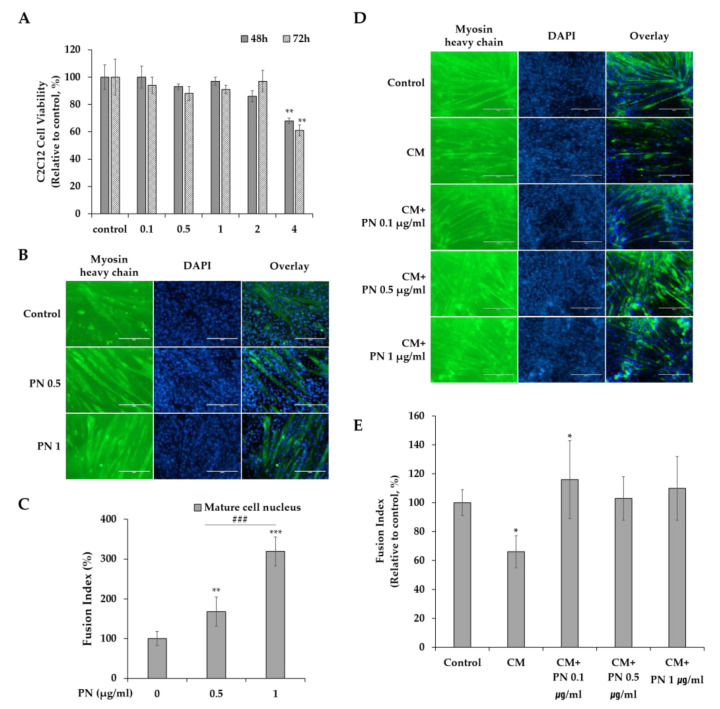
(**A**) C2C12 cells were treated with different PN concentrations (0, 0.1, 0.5, 1, 2 and 4 µg/mL) for 48 and 72 h. Results are presented as means ± S.D. of 3 independent experiments, * *p* < 0.05, ** *p* < 0.01, *** *p* < 0.001. (**B**) Immunofluorescence microscopy for the expression of the myogenic markers Myosin heavy Chain (MyHC) and DAPI. Myogenesis was induced in differentiation media and treated for 5 days with different PN concentration (0, 0.5 and 1 µg/mL). (**C**) Fusion indexes were calculated as the % of the nuclei inside myotubes compared to the total number of nuclei. (N= 3 independent experiments; mean SEM, * *p* < 0.05, ** *p* < 0.01, *** *p* < 0.001; Student’s *t* test). Between experimental groups, statistical difference was found via one-way ANOVA (### *p* < 0.001). (**D**) Representative images of myotube formation on cancer conditioned media upon PN treatment (0, 0.1, 0.5 and 1 μg /mL). Myotube was detected with MyHC (green) immunostaining and nuclear counterstained DAPI (blue). Scale bar = 200 μm. (**E**) Fusion index were calculated as the % of the nuclei inside myotubes compared to the total number of nuclei. (Data were from three independent experiments; mean SEM, * *p* < 0.05; Student’s *t* test).

During cancer progression, skeletal muscles are weakened which causes progressive functional impairment, called cachexia [21,22,23]. Accordingly, it is important aspects that agents targeting cancers do not adversely affect muscle cells. Thus, we examined the proliferation and function of muscle cells of myoblast, treated with different concentrations of PN (0, 0.1, 0.5, 1, 2, or 4 µg/mL) for 48 or 72 h. In Figure 6A, PN did not inhibit cell proliferation up to 2 µg/mL concentrations. After 72 h of treatment with 2 or 4 µg/mL of PN, cell viabilities remained at 97% and 61% of non-treated control levels. Next, we examined if PN affected muscle functions of myotube formation by inducing myogenic differentiation. Phenotypes were detected by myosin heavy chain (MyHC) immunostaining and fusion indices were calculated. Surprisingly, PN treatment did not inhibit myogenic differentiation as determined by MyHC staining and fusion indices (Figure 6B,C). In fact, fusion indices even improved at all PN treated groups. Our data suggest that PN has a beneficial effect on muscle cell function. 

Because PN did not impair and rather supported C2C12 myogenic differentiation into myotube formation, we further investigated if PN could attenuate conditioned media (CM)-mediated muscle function impairment. In Figure 6D,E, reduced myotube numbers and MyHC expression were observed on PN-untreated control, which indicated cancer environment significantly impaired muscle cell function. However, PN-treatment rescued C2C12 muscle cell dysfunctions. In detail, increased myotube formation and MyHC expression compared to the SW480 control CM were observed (Figure 6D,E).

During cancer progression, cancer-associated cachexia is common [21], and one of the problems posed by anti-cancer drugs is that they impair normal cell proliferation and function. Therefore, the maintenance of muscle cell proliferation and myogenic differentiation are important aspects at the cancer therapeutic development. Interestingly, we found PN did not impair muscle cell proliferation, but actually restored muscle cell function.

## 3. Materials and Methods

### 3.1. Preparation of PN from the Seeds of Pharbitis nil

PN was prepared as previously reported [34]. In brief, dried seeds of *Pharbitis nil* (1 kg) were ground. Then, ground *Pharbitis nil* was immersed in methanol (MeOH) at room temperature for 48 h and extracted. The MeOH extract was filtered with 5 µm filter paper and concentrated using a rotary evaporator (Tokyo Rikakikai, Tokyo, Japan). To purify compound PN, the MeOH extract (5 g) was dissolved in 50% MeOH and chromatographed on a Diaion HP-20 column by the gradient MEOH (50%, 80%, 100%, each fraction 500 mL). The 100% MeOH fraction (800 mg) was further chromatographed on reversed-phase C-18 and Sephadex LH-20 column using 100% MeOH as mobile phase, simultaneously. The 250 mg of purified compound PN was confirmed by high performance liquid chromatography (HPLC) in our previous study.

### 3.2. Cell Culture and Treatment

The human CRC cell-lines, SW480, HCT116, HT29 and WiDR were purchased from the American Type Culture Collection (Rockville, MD, USA). G12V KRAS mutated SW480 (KRAS^G12V^), G13D KRAS mutated HCT116 (KRAS^G13D^), KRAS wild types of HT29 and WiDR were cultured in RPMI-1640 supplemented with 10% fetal bovine serum (FBS; Invitrogen), and 1% penicillin-streptomycin (P/S; Sigma, St. Louis, MO, USA). C2C12 cells were purchased from ATCC and cultured in DMEM (Dulbecco’s modified Eagle’s medium) containing 10% FBS and 1% P/S in a 5% CO_2_ atmosphere at 37 °C.

### 3.3. Cell Proliferation by MTS Assay

An MTS (3-(4,5-dimethylthiazol-2-yl)-5-(3-carboxymethoxyphenyl)-2-(4-sulfophenyl)-2H-tetrazolium) assay (Promega, Fitchburg, WA, USA) was used to determine the anti-proliferative effect of PN on CRC cell lines and its toxic effect on C2C12 muscle cells. For each cell line tested, 2 × 10^4^ cells per well were seeded onto 96-well plates and allowed to attach. Cells were treated with PN at 0.1, 0.5, 1, 2, or 4 µg/mL and incubated for 48 and 72 h. Cetuximab of 30 µg/mL was added as a control. MTS solution (20 μL) was then added and cells were incubated at 37 °C for 1 h. Absorbance were measured at 490 nm (Model 550, Bio-Rad, Hercules, CA, USA). The experiments were performed in triplicate. IC_50_ values (concentrations that inhibited cell growth by 50%) were compared. 

### 3.4. DAPI/PI Staining

Cells were treated with PN for 48 h, fixed with 4% paraformaldehyde (Sigma), washed with PBS, and stained with DAPI (4′,6-diamidino-2-phenylindole, Sigma) to detect cell nuclei and with PI (propidium iodide, Sigma) to detect dead cells in the dark. Stained cells were then observed under a fluorescence microscope (EVOS®, Thermo Scientific, Waltham, MA, USA). 

### 3.5. Clonogenic Assay

Human CRC cell lines were seeded at 1 × 10^3^ cell/well onto six-well plates. After cells had completely attached, they were treated with 1 or 4 µg/mL of PN, and 10 days later, fixed with 4% paraformaldehyde for 10 min, dried, and stained with 0.05% crystal violet (Sigma) for 15 min. The experiments were performed in triplicate.

### 3.6. Cell Apoptosis Analysis

Human CRC cells were treated with PN at 0.1, 0.5, 1, 2, or 4 µg/mL for 48 h, harvested, stained with Annexin V-FITC and PI for 15 min in the dark, according to the manufacturer’s instructions (BD Biosciences, San Jose, CA, USA). Apoptotic cells were analyzed by flow cytometry (FC 500, Beckman Coulter, Indianapolis, IN, USA). The experiments were performed in triplicate.

### 3.7. Cell Cycle Analysis

Cell cycles were analyzed by flow cytometry in triplicate. Cells were seeded, treated with PN at 0.5, 1, 2, or 4 µg/mL for 24 h, detached, fixed in ice-cold 70% ethanol, and stained with PI solution containing RNase for 10 min in the dark. Cell cycle distributions were analyzed by flow cytometry (FC 500). 

### 3.8. Western Blotting

Western blot analysis was performed in triplicate as previously described [14]. Cells were treated with compound PN (0.5–4 µg/mL) for 48 h and detached with trypsin-EDTA. Proteins were isolated with RIPA buffer containing protease inhibitors (Sigma) and quantified using a bicinchoninic acid (BCA) assay (Thermo Scientific). Proteins were separated in precast SDS-PAGE gels (10%, Invitrogen) and transferred to membranes using the iBlot system (Thermo Scientific). Membranes were blocked with 5% skimmed milk in Tris buffered saline (TBS) and incubated with the following primary antibodies overnight at 4 °C; cdc2, cyclin B1, HSP90, phospho-mTOR, GAPDH, KRAS, phospho-ERK1/2, and phospho-AKT. Membranes were then washed and incubated with HRP-conjugated goat anti-rabbit or anti-mouse antibodies (Pierce, Rockford, IL, USA). Densitometric analysis was performed using ImageJ software (NIH, Bethesda, MD, USA) and ratios of proteins versus the loading control were calculated. 

### 3.9. Myogenic Differentiation

C2C12 myoblasts were seeded onto six-well plates (4 × 10^4^ cells/well) in triplicate and cultured for 2 days in DMEM. Myogenesis was induced by culturing cells in differentiation medium (DMEM, 2% Horse serum, 1% P/S) containing PN at 0.5, 1 µg/mL for 5 days [35]. Differentiated muscle cells were immunostained for fast myosin heavy chain (f-MyHC, a marker of terminal myogenic differentiation), fixed with 4% paraformaldehyde, blocked with 5% goat serum, and incubated at room temperature with myosin 4 monoclonal antibody (MF20)-Alexa Fluor 488 (Thermo Scientific) overnight at 4 °C. The nuclei were visualized by staining with DAPI (Sigma) for 10 min. Total cell nuclei and nuclei within f-MyHC-positive myofibers in at least 15 fields of three replicate platings were counted by using ImageJ. Myotube fusion indices were calculated by expressing the number of cells expressing f-MyHC (green) as a percentage of total nuclei (DAPI, blue) [35,36]. 

### 3.10. Preparation of SW480-Conditioned Media

To prepare conditioned media, SW480 cells were cultured on the 100 mm cell culture dish with the concentration of 5 × 10^4^ cells/seeding. After 24 h, cells were washed with 1× PBS and changed to serum-free DMEM. Upon one day of incubation, media were collected and supernatant parts were filtered through 0.2 μm syringe filter for conditioned media (CM). 

### 3.11. C2C12 Myogenic Differentiation in Cancer Conditioned Media 

To investigate the effect of PN on C2C12 myogenic differentiation under during cancer progression, conditioned media (CM) were applied as follows. First of all, C2C12 myoblasts were seeded onto six-well plates (4 × 10^4^ cells/well) and cultured for 2 days in regular DMEM. After cells are attached, the mixture of CM and differentiation media (2% horse serum in DMEM) with the ratio of 1:3 were added and PN with the concentrations of 0.1, 0.5 and 1 µg/mL were treated.

### 3.12. Statistical Analysis

Results were reported as means ± standard errors of means (SEM) of experiments performed in triplicate. Comparisons among experimental groups were performed by one-way ANOVA using SPSS ver. 12.0 (SPSS, IBM, USA). *p*-values of < 0.05 were deemed significant.

## 4. Conclusions

The results of the present study showed two beneficial effect of PN on KRAS-mutated CRC. Firstly, it significantly suppressed KRAS-mutated CRC progression. Cell proliferation and clonogenic potential were decreased at relatively low concentrations via apoptosis and G2/M phase arrest. The mechanism responsible for the effects of PN may be associated with the AKT/mTOR signaling pathway. Secondly, PN rescued muscle cells dysfunction under cancer progression. Collectively, our results provide support for the incorporation of PN into therapeutic regimens targeting mutated KRAS-driven CRC. For future research, the toxicity and efficacy of PN needs to be performed in animal studies.
